# MAXIMUM HEART RATE MEASURED VERSUS ESTIMATED BY DIFFERENT EQUATIONS DURING THE CARDIOPULMONARY EXERCISE TEST IN OBESE ADOLESCENTS

**DOI:** 10.1590/1984-0462/;2018;36;3;00015

**Published:** 2018

**Authors:** João Paulo Heinzmann-Filho, Letiane Bueno Zanatta, Fernanda Maria Vendrusculo, Juliana Severo da Silva, Mailise Fatima Gheller, Natália Evangelista Campos, Margareth da Silva Oliveira, Ana Maria Pandolfo Feoli, Andréia da Silva Gustavo, Márcio Vinícius Fagundes Donadio

**Affiliations:** a Pontifícia Universidade Católica do Rio Grande do Sul, Porto Alegre, RS, Brasil.

**Keywords:** Heart rate, Cardiovascular physiological phenomena, Exercise test, Obesity, Pediatrics, Adolescent, Frequência cardíaca, Fisiologia cardiovascular, Teste de esforço, Obesidade, Pediatria, Adolescente

## Abstract

**Objective:**

To compare the values of measured maximum heart rate (HRmax) and maximum heart rate estimated by different equations during the cardiopulmonary exercise test (CPET) in obese adolescents.

**Methods:**

This is a cross-sectional study. Adolescents aged between 15 and 18 years old, with obesity (BMI Z-score>2.0) were included. Demographic and anthropometric data were collected, followed by CPET, recording HRmax. The highest heart rate reached at peak exercise was considered as HRmax. The comparison between measured and estimated HRmax values was performed using four previous equations. Descriptive statistics and the ANOVA test (Bonferroni post-test) were used.

**Results:**

Fifty-nine obese adolescents were included, 44% of them male. The mean age was 16.8±1.2 years old and the BMI (Z-score) was 3.0±0.7. At peak exercise, the mean HRmax (bpm) was 190.0±9.2, the respiratory coefficient was 1.2±0.1, and the VO_2_max (mL/kg/min) was 26.9±4.5. When comparing the measured values of HRmax with those estimated by the different formulas, the equations “220-age”, “208-0.7 x age” and “207-0.7 x age” were shown to overestimate (p<0.001) the measured HRmax results in obese adolescents. Only the “200-0.48 x age” equation presented similar results (p=0.103) with the values measured in the CPET.

**Conclusions:**

The findings of the present study demonstrate that the equation “200-0.48 x age” seems to be more adequate to estimate HRmax in obese adolescents.

## RESUMO

**Objetivo:**

Comparar os valores de frequência cardíaca máxima (FC_máx_) medidos e estimados por diferentes equações durante o teste de exercício cardiopulmonar (TECP) em adolescentes obesos.

**Métodos:**

Trata-se de um estudo transversal. Foram incluídos adolescentes, de idades entre 15 e 18 anos, com obesidade (escore-Z do índice de massa corpórea - IMC>2,0). Coletaram-se dados demográficos e antropométricos, seguidos da realização do TECP, pela qual foi registrada a FC_máx_. O valor mais elevado de frequência cardíaca (FC) atingida no pico do exercício foi considerado como a FC_máx_. A comparação entre os valores de FC_máx_ medidos e os estimados pelas equações foi realizada empregando-se quatro equações prévias. Utilizaram-se a estatística descritiva e o teste de ANOVA (pós-teste de Bonferroni).

**Resultados:**

Foram incluídos 59 adolescentes obesos, sendo 44% do sexo masculino. A média de idade foi de 16,8±1,2 anos e a do IMC (escore-Z), de 3,0±0,7. No pico do exercício, a média de FC_máx_ (batimentos por minuto - bpm) foi de 190,0±9,2, o coeficiente de troca respiratória de 1,2±0,1 e o consumo máximo de oxigênio - VO_2máx_ (mL/kg/min) - de 26,9±4,5. Ao comparar-se os valores medidos de FC_máx_ com os estimados pelas diferentes fórmulas, demonstrou-se que as equações “220-idade”, “208-0,7 x idade” e a “207-0,7 x idade” superestimam (p<0,001) os resultados medidos de FC_máx_ em adolescentes obesos. Apenas a equação “200-0,48 x idade” apresentou resultados similares (p=0,103) com os valores mensurados no TECP.

**Conclusões:**

Os achados do presente estudo demonstram que a equação “200-0,48 x idade” parece ser mais adequada para estimar a FC_máx_ em adolescentes obesos.

## INTRODUCTION

Heart rate (HR) is an easy-to-measure physiological parameter commonly used to assess cardiovascular responses during exercise and recovery phases.^1^ ^,^ ^2^ HR increases linearly with exercise intensity, and maximum heart rate (HRmax) is the highest value an individual achieves during physical exercise, when they reach a point of exhaustion.^3^

HRmax is used as an important indicator for the prescription of exercise intensity, due to its relation to maximum oxygen consumption (VO_2max_), in the range between 50 and 90% VO_2max_.^2^ ^,^ ^4^ Generally, untrained individuals have high HR values both in rest and maximal physical exertion states, when compared to trained individuals.^5^ ^,^ ^6^ Data also indicate that physical training causes reduced HRmax as a result of cardiac pump and autonomic nervous system adaptations that are made in order to achieve an efficient cardiac output.^7^ In addition, elevated HR at rest is considered to be an independent predictor of mortality in the general population and in subjects with cardiovascular diseases.^5^ ^,^ ^8^

HRmax can be determined directly, through ergometers with incremental effort protocols, or indirectly, through predictive equations.^1^ ^,^ ^9^ ^,^ ^10^ Some formulas for predicting HRmax from studies with adult populations^9^ ^,^ ^11^ have already been tested in child populations,^1^ ^,^ ^12^ ^,^ ^13^ including the evaluation of healthy participants, and participants with previous diseases. In general, it seems that the classical “220-age” equation^11^ tends to overestimate the measured values of HRmax in the pediatric age group.^13^ ^,^ ^14^ Other equations or cut-off points have been suggested in order to estimate this variable in samples with young individuals.^9^ ^,^ ^15^ To our knowledge, no study has investigated how HRmax works in obese adolescents. It is possible that predictive equations could compromise studies performed with the infant population, considering the possible influence of nutritional status on cardiovascular performance.

Therefore, considering how well HRmax is used for evaluation, prescription and clinical prognosis, in addition to the lack of information on the most representative predictive equation for the obese adolescent population, the present study was developed. Thus, the objective of this research was to compare the measured and estimated values of HRmax using different equations during the cardiopulmonary exercise test (CPET) in obese adolescents.

## METHOD

This is a cross-sectional study. Adolescents, aged 15 to 18 years old, with obesity (body mass index Z scores - BMI>2.0) were included. Conversely, participants with musculoskeletal, neurological, vascular, pulmonary and cardiac problems were excluded. Additionally, individuals who were unable to meet the exhaustion criteria in the CPET were excluded.

This study is part of an umbrella research project called “The Effects of Interdisciplinary Intervention with a Motivational Approach to Lifestyle Modification on Overweight and Obese Adolescents.” This study was approved by the Research Ethics Committee of the Pontifícia Universidade Católica do Rio Grande do Sul (PUCRS) under CAAE No. 36209814.6.0000.5336 in accordance with the Declaration of Helsinki. All parents ­and/­or legal guardians signed a free and informed consent form, and the adolescents also signed a consent form.

Participants were selected by convenience through announcements and invitation letters. Data collection was performed at the São Lucas Hospital (*Hospital São Lucas* - HSL) of PUCRS, during the morning and afternoon shifts, from August 2014 to December 2016. Demographic (age and gender) and anthropometric data (weight, height and BMI) were collected, followed by CPET and its physiological variables (cardiovascular, ventilatory, metabolic and subjective). All evaluations were performed by trained researchers, prior to the inclusion of participants in the umbrella intervention program.

The anthropometric measurements were evaluated by recording the individual’s weight and height three times, or until two identical values were obtained. Weight was obtained with the individuals in an orthostatic position, wearing as little clothing as possible, no shoes, and using a digital scale (G-Tech, Glass 1 FW, Rio de Janeiro, Brazil) with 100 g precision. Height was collected while the participants were barefoot, and their feet were placed in a parallel position with their ankles touching. Height measurements were obtained using a portable stadiometer (AlturaExata, TBW, São Paulo, Brazil) with an accuracy of 1 mm. From these measurements, the anthropometric characteristics were normalized by means of the Z-score for BMI.^16^

The cardiopulmonary exercise test was performed according to recommendations from the American Thoracic Society and the American College of Chest Physicians.^17^ All tests were performed at room temperature between 22 and 24°C, and with relative air humidity around 60%. The evaluation was performed using a computerized system (Aerograph, AeroSport^®^, United States), coupled to a gas analyzer (VO_2000_, MedGraphics^®^, USA), and using a treadmill (KT-10400, Inbramed^®^, Brazil). The variables collected included VO_2max_, maximum ventilation (VEmax), respiratory coefficient (RQ), peripheral oxygen saturation (SpO_2_), subjective levels of dyspnea and fatigue in the legs (modified Borg scale),^18^ pulse oxygen (VO_2_/HR) and HRmax. HRmax was obtained with the aid of a pulse oximeter (DX2405, Dixtal^®^, Brazil), using the highest value that an individual can reach when putting in maximum effort, to the point of exhaustion. The test was performed with a ramp protocol, which was adapted according to a previous study.^19^ Participants were instructed to walk for two minutes to adapt to the treadmill, at a speed of 3 km/h, with no inclination. Thereafter, velocity was increased by 0.5 km/h per minute, with a steady slope of 3% until the test was completed.^19^ Everyone was encouraged to keep up the pace until they were completely exhausted, or until limiting signs and/or symptoms (dyspnea, leg pain ­and/­or dizziness) appeared. In order for the test to be considered a maximum, at least three of the following criteria had to be observed: exhaustion or inability to maintain the required velocity, respiratory coefficient> 1.10, HRmax> 85% of estimated HR (formula: 220-age) and a plateau in VO_2max_.^20^ ^,^ ^21^

The comparison between the measured values of HRmax and the values estimated by the equations was performed using four previously determined equations. These formulas were named according to their prediction equation to make reading the data easier. Thus, the equations “220-age”,^11^ “208-0.7 x age”,^9^ “207-0.7 x age”^22^ and “200-0.48 x age” were identified.

The sample calculation was estimated based on data from the first 25 participants of the study, considering the variability of measured HRmax and HRmax estimated by the equation “220-age”. Thus, considering a standard deviation of 8.9 for measured HRmax and 1.14 for estimated HRmax, a minimum difference to be detected of 4 beats per minute (bpm), a significance level of 0.05 and a power of 90%, a sample size of 55 participants was estimated.

The main variables of the study were evaluated using the Kolmogorov Smirnov test. The data that had normal distribution were presented using mean and standard deviation, while the asymmetric data, were presented using median and interquartile range. The comparisons of the measured HRmax values, with the results estimated by the different prediction formulas and with the influence of age on this variable, were performed using the one-way ANOVA test (Bonferroni post test). All analyzes and data processing were performed using the Statistical Package for the Social Sciences (SPSS) version 18.0 (SPSS Inc., USA). In all of the cases, differences were considered to be significant when the p-value<0.05.

## RESULTS

Of a total of 61 participants, two were excluded because they did not meet the CPET exhaustion criteria. Thus, 59 obese adolescents were included, 44% of them, male. The mean age was 16.8±1.2 years old, with a BMI (Z-score) of 3.0±0.7. Table 1 shows the demographic and anthropometric data of the sample.

**Table 1:** Characteristics of the 59 evaluated adolescents.

Evaluated variables	Mean/frequency	Variation (minimum- maximum)
Demographic		
Age, years (mean±standard deviation)	16.8±1.2	15.0-18.8
Male [n (%)]	44.0 (74.6)	-
Anthropometric		
Height, cm (mean±standard deviation)	165.0±8.1	137.5-184.0
Weight, kg (mean±standard deviation)	97.3±17.1	56.9-141.2
BMI, absolute (mean±standard deviation)	35.6±4.7	29.1-48.3
BMI, Z-score (mean±standard deviation)	3.0±0.7	2.0-4.8

BMI: body mass index.

With regard to the CPET results at peak exercise, the mean HRmax was 190.0 bpm (93.5% predicted HRmax), with a RQ of 1.2, a VO_2_ of 26.9 mL/kg/min and a VE_max_ of 68.5 L/min. The majority of the subjects requested the test to be stopped due to dyspnea and/or leg fatigue (median of 5), indicating that the adolescents reached the test’s exhaustion criteria. Table 2 shows the results of the physiological variables obtained from the CPET.

**Table 2:** Results of the cardiopulmonary exercise test in the 59 obese adolescents evaluated.

Variables evaluated	Mean/median	Variation (minimum-maximum)
Cardiovascular		
HRmax, bpm	190.0±9.2	166.0-209.0
SpO_2_, %	96.0±2.0	90.0-99.0
VO_2_/HR, mL/min/beat	15.9±6.5	10.8±41.7
Ventilation		
VO_2_, L/min	2.6±0.6	1.6-4.9
VO_2_, mL/kg/min	26.9±4.5	18.0-36.4
VE_máx_, L/min	68.5±17.8	35.0-129.4
Metabolic		
RQ	1.2±0.1	1.1-1.4
VO_2_ at AT, mL/kg/min	21.5±6.2	11.0±38.9
Subjective		
Borg for dyspnea	5.0 (3.2-7.0)	0.0-10.0
Borg for lower limbs	5.0 (3.0-7.0)	0.0-10.0

Continuous data presented in mean ± standard deviation, except for the Borg variable (median [interquartile range]); categorical variables expressed in absolute and relative frequency; SpO_2_: peripheral oxygen saturation; VO2: maximum oxygen consumption; HR: heart rate; bpm: beats per minute; VEmax: maximum ventilation; RQ: respiratory coefficient; AT: anaerobic threshold.

**Figure 1:**
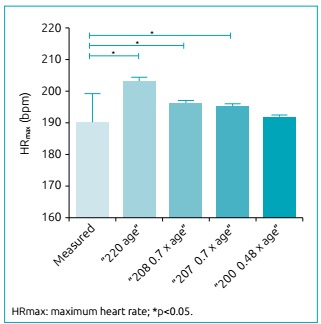
A comparison between the measured values of maximum heart rate (bpm) and the values estimated by the different formulas in obese adolescents.

When comparing measured values of HRmax (bpm) with those estimated by the different formulas, it was shown that the “220-age”, “208-0.7 x age” and “207-0.7 x age” equations overestimated (p<0.001) the measured HRmax results in the sample. These equations tend to overestimate, on average, 13.2 bpm [estimated HR: 203.2±1.2], 6.3 bpm [estimated HR: 196.3±0.8] and 5.3 bpm [Estimated HR: 195.3±0.8] the HRmax reached by the adolescents, respectively. Only the “200-0.48 x age” equation presented similar results (p=0.103) with the values measured in the study, recording only 1.9 bpm [estimated HR: 191.9±0.6] in addition to the measured result (Figure 1).

On the other hand, there was no significant difference (p=0.164) in the comparison of HRmax values measured between the different ages (15 to 18 years old). The results of HRmax (bpm) according to age were 188.0±11.4 (Δ_HR_: 43), 192.2±7.0 (Δ_HR_: 25), 193.4±5.6 (Δ_HR_: 18) and 186.9±9.5 (Δ_HR_: 35), respectively.

**Figure 2:**
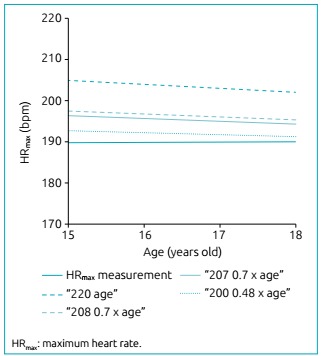
Scatter plot of the maximum measured heart rate and the values estimated by the different equations in relation to age.

Finally, Figure 2 shows a scatter plot, showing the measured HRmax performance and the values estimated by the different equations in relation to age. Again, the data indicate that the equation “200-0.48 x age” remains closer to the values measured along the assessed age range.

## DISCUSSION

The HRmax prediction equation directly implies the amount of effort the cardiovascular system requires during exercise. Thus, important biases in estimating this can lead to failures in the prescription of exercise intensity, as well as in the success of results achieved after the training. The findings of the present study demonstrate that the prediction of HRmax in obese adolescents should be performed from the equation “200-0.48 x age”, considering that the measured values and those estimated by this equation were the most similar.

To our knowledge, this is the first study to test different predictive equations of HRmax in obese adolescents, taking into account the possible effect of body mass on cardiovascular performance. Our findings corroborate the fact that the predictive equations are adequate when applied in populations with characteristics similar to those in the sample from which the formula was created. Although the equations used in the present study were generated from samples with adult subjects,^9^ ^,^ ^11^ ^,^ ^22^ ^,^ ^23^ only the equation “200-0.48 x age” was generated based on obese individuals,^23^ which is close to the characteristics presented in this study, justifying the results found. An interesting fact is that this study^23^ reports that it is necessary to use a specific equation for the obese population, while suggesting that the “220-age” equation for eutrophic adults may continue to be used.

Although the classic “220-age” equation^11^ has often been used as an important feature of Exercise Physiology since the 1930s, it appears that the equation was not generated from original research, as reported in a previous publication.^2^ Such an equation was created from the observation of 11 studies published in and out of the field of Physiology, which compromises its scientific validation in the field of exercise.^2^ Nevertheless, several studies have continued to test the use of this formula to estimate HRmax in the pediatric and adult population.^1^ ^,^ ^3^ ^,^ ^13^ As such, there seems to be a consensus that this equation overestimates the measured HRmax results in the pediatric population,^1^ ^,^ ^13^ ^,^ ^24^ which corroborates our results. Moreover, a previous report^23^ indicates that this equation seems to overestimate the results of HRmax in samples of people under the age of 40 years old, and underestimate the findings in elderly people.

The results of the present study also showed that there was no significant difference in HRmax measured between the age groups evaluated, and that there is variability for it when we observed the standard deviation/delta of our results. This variation (standard deviation) ranged from 5.6 to 11.4 bpm in the sample, which is close to the values reported by previous research.^1^ ^,^ ^2^ A recent study with pediatric athletes reported that age did not significantly influence the prediction model to estimate HRmax, as other variables were included such as resting HR, physical fitness, body mass, and fat percentage.^15^ In addition, the study suggests that a cut-off point of at least 180 bpm should be used to estimate HRmax in children and adolescents due to the small predictive power of their formula, and the fact that the measured values are in a similar range.^15^ As such, there has been suggesting evidence that age does not significantly influence HRmax in pediatric samples, which could be justified by the different chronotropic effects of this and the weak correlation between age and HRmax in young people.^25^

The present study also tested two other HRmax predictive equations^9^ ^,^ ^22^: “208-0.7 x age”^9^ and “207-0.7 x age.” ^22^ However, both of them overestimated exercise peak HR, estimating, on average, 6.3 and 5.3 bpm beyond the observed value, respectively. Such equations have been previously tested in other studies,^1^ ^,^ ^3^ ^,^ ^15^ ^,^ ^26^ ^,^ ^27^ ^,^ ^28^ ^,^ ^29^ and some were conducted with pediatric populations^1^ ^,^ ^15^ ^,^ ^29^ and others with samples from adult individuals.^3^ ^,^ ^26^ ^,^ ^28^ In large part,^3^ ^,^ ^27^ ^,^ ^28^ the “208-0.7 x age” equation appears to be adequate for the adult population, while only one study reported its validity for infant samples,^1^ differing from our findings. However, such results can be explained because this study included only healthy and active boys (10 to 16 years old),^1^ which is different from the sample characteristics of the present study. In addition, another study^15^ demonstrated that there is a weak correlation between measured HRmax in a pediatric sample and values predicted by the “207-0.7 x age” equation, indicating little validity when applied to children and adolescents.

One of the limitations of the present study was the restricted age group being evaluated, considering that individuals under the age of 15 were not included, which could have confirmed these results with younger aged children. However, our findings indicate that the “200-0.48 x age” equation, derived from a sample of obese adults, should be the first choice for training or rehabilitation centers at the time of predicting HRmax in obese adolescents. Hopefully, future studies will test the usefulness of this equation in samples composed of younger children and adolescents, considering that CPET is usually performed starting at the age of eight years old.

In conclusion, the findings of the present study demonstrated that the “200-0.48 x age” equation seems to be more adequate to estimate HRmax in obese adolescents, indicating the importance of using specific equations for each sample investigated. These results present an important clinical relevance, considering that this variable is used both in the prescription of exercises for weight reduction and improvement of physical fitness, as well as in the monitoring of physical activity. Overall, the goal is to exercise safely in order to avoid possible adverse events.
